# The role of genetic variants in the prediction of hearing loss due to cisplatin chemoradiotherapy

**DOI:** 10.1002/cam4.7465

**Published:** 2024-08-19

**Authors:** Charlotte W. Duinkerken, Sabrina Chiodo, Katrina Hueniken, Michael Hauptmann, Katarzyna Jóźwiak, Dangxiao Cheng, Andrew Hope, Geoffrey Liu, Charlotte L. Zuur

**Affiliations:** ^1^ Department of Otolaryngology and Head and Neck Surgery Leiden University Medical Centre Leiden the Netherlands; ^2^ Department of Head and Neck Surgery the Netherlands Cancer Institute Amsterdam the Netherlands; ^3^ Dalla Lana School of Public Health University of Toronto Toronto Canada; ^4^ Department of Medical Oncology and Hematology Princess Margaret Cancer Centre Toronto Canada; ^5^ Department of Biostatistics University Health Network Toronto Canada; ^6^ Institute of Biostatistics and Registry Research Brandenburg Medical School Theodor Fontane Neuruppin Germany

**Keywords:** cisplatin, head and neck squamous cell carcinoma, hearing loss, *MATE1*, SNP

## Abstract

**Background:**

Concomitant high‐dose cisplatin with radiotherapy is commonly used for treating head and neck squamous cell carcinoma (HNSCC). Cisplatin, often used with radiotherapy, is known for causing irreversible sensorineural hearing loss, with individual variability suggesting a genetic component. This study aims to enhance the predictive ability of the clinical prediction model for cisplatin‐induced hearing loss (CIHL) in HNSCC patients, as outlined in Theunissen et al., by incorporating significant genetic variants.

**Methods:**

Conducted at the Netherlands Cancer Institute, this retrospective study included 74 patients treated between 1997 and 2011. Thirty‐one SNPs that were previously associated with CIHL or other cisplatin‐induced toxicities were identified and incorporated into the model. The primary outcome measured was the change in decibels at posttreatment 1‐2‐4 kHz hearing levels per additional minor allele of these SNPs, evaluated using linear mixed‐effects regression models. The model's predictive accuracy was determined by the area under the curve (AUC) using 10‐fold cross‐validation.

**Results:**

The rs2289669 SNP in the *SLC47A1/MATE1* gene was linked to a significant 2.67 dB increase in hearing loss per allele (95% CI 0.49–4.86, *p* = 0.017). Incorporating rs2289669 improved the model's AUC from 0.78 to 0.83, a borderline significant improvement (*p* = 0.073).

**Conclusions:**

This study underscores the importance of the rs2289669 SNP in CIHL and demonstrates the potential of combining genetic and clinical data for enhanced predictive models in personalized treatment strategies.

## Graphical abstract

This retrospective study conducted at the Netherlands Cancer Institute evaluated the addition of genetic variants to enhance a predictive model for cisplatin‐induced hearing loss in patients with head and neck squamous cell carcinoma. The findings highlighted the significance of the rs2289669 SNP in the SLC47A1/MATE1 gene, which was associated with a notable increase in hearing loss per allele. Incorporating this SNP into the model improved its predictive accuracy, emphasizing the value of integrating genetic and clinical data for personalized treatment strategies.

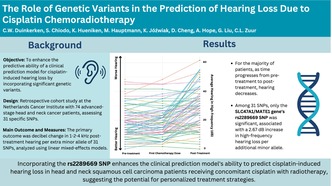

## 1 INTRODUCTION

Cisplatin is a widely used chemotherapeutic agent for patients with advanced head and neck squamous cell carcinoma (HNSCC), who are treated with concurrent chemoradiotherapy.^1^ Dose‐limiting side effects of cisplatin include nephrotoxicity, neurotoxicity, and ototoxicity.^2^, ^3^, ^4^ There is no standardized protective or curative agent available for neuro‐ and ototoxicity.^2^ However, there is an increasing interest in the search for otoprotective agents, including amifostine, dexamethasone, and vitamin E, with varying success.^5^, ^6^, ^7^, ^8^, ^9^ Recently, trans‐tympanic sodium thiosulfate has also been studied as an otoprotector in a phase I study at the Netherlands Cancer Institute (NKI) and in a meta‐analysis.^5^, ^10^

Cisplatin has been known to instigate cochlear dysfunction, leading to sensorineural hearing loss (SNHL). According to a recent meta‐analysis, the prevalence of cisplatin‐induced hearing loss (CIHL) is 49% in adults with HNSCC,^8^ although the incidence of CIHL varies across studies due to differing definitions and cisplatin doses.^2^, ^3^, ^11^, ^12^, ^13^ CIHL is characterized by symmetric and irreversible SNHL starting at high frequencies, but after continued cisplatin courses it may progress to lower frequencies involved in speech perception.^2^, ^14^, ^15^ Other risk factors for the development of CIHL include favorable pretreatment hearing capacity, the use of other ototoxic drugs, and radiation exposure of the cochlea.^8^, ^9^, ^12^, ^16^, ^17^ Also, genetic variants have been associated with CIHL, such as single‐nucleotide polymorphisms (SNPs) including *ACYP2*,^18^, ^19^, ^20^, ^21^, ^22^ *WFS1*,^18^, ^22^ *ABCC3*,^23^, ^24^ and *MATE1*.^25^

Theunissen and colleagues developed a prediction model for posttreatment hearing loss in patients with HNSCC who are treated with combined chemoradiation, see Figure S1.^26^ This model uses clinical input variables, such as baseline hearing thresholds, cisplatin dose, and cochlear radiation dose, to predict posttreatment hearing thresholds averaged at frequencies 1, 2, and 4 kHz. The model shows a 97% specificity and 29% sensitivity for the indication of hearing aids in the Netherlands (35 dB threshold).^26^ Despite the model being a significant step toward improving individual recommendations for HNSCC patients at risk for CIHL, the authors called for future research concerning additional risk factors. In particular, they hypothesized the role of genetic variants in hearing loss severity, based on observations of substantial individual vulnerability for the prevalence and severity of CIHL.^26^, ^27^ Identifying SNPs linked to CIHL is crucial for personalized care, enabling better pretreatment guidance and interventions against chemoradiation side effects, including the use of trans‐tympanic sodium thiosulfate for prevention.

The main aim of this study is to describe the contribution of genetics to individualized susceptibility to CIHL in an extension cohort of Theunissen et al.^26^ and investigate whether using SNPs that are significantly associated with CIHL increases the predictive ability of our previously designed clinical prediction model.^26^

## 2 METHODS

### 2.1 Data sources and patient selection

A retrospective cohort study was performed at the NKI from patients treated with intravenous (IV) cisplatin (100 mg/m^2^, for three courses) or with intra‐arterial (IA) cisplatin (150 mg/m^2^; for four courses, within the “RADPLAT trial”)^28^ during 7 weeks of radiotherapy (total dose of 70 Gy in 35 fractions on tumor‐bearing areas) for advanced‐stage HNSCC. All patients were treated between January 1, 1997 and December 31, 2011. Patients were selected based on available pre‐ and posttreatment audiometry and formalin‐fixed paraffin‐embedded tissue blocks. The patients treated with IA cisplatin have also been treated with concurrent IV sodium thiosulphate in order to prevent from platinum‐related toxicity. We chose to also include these patients in the current cohort, as in these IA treated patients sodium thiosulphate did not protect against CIHL.^28^ The cochlear radiotherapy dose was also measured (methods described by Zuur et al.^17^). All patients gave informed consent for further use of their data.

### 2.2 Audiometry

Pure‐tone audiometry was conducted pretreatment, 15–20 days after the first cisplatin infusion, and 3–31 weeks posttreatment in a sound‐proof booth using the Decos Audiology Workstation. Audiometry consisted of pure tone audiometry (in hearing level (HL)) from 0.125 to 8 kHz, including both air conduction (AC) and bone conduction (BC), and ultrahigh frequency audiometry (in sound pressure level (SPL)) from 8 to 20 kHz. BC thresholds were used for the frequencies 0.5, 1, 2, and 4 kHz to correct for potentially fluctuating conductive components. Three pure tone averages (PTAs) were calculated: for the relatively low‐frequency range relevant to quiet speech perception, we calculated PTA 0.5‐1‐2 kHz HL BC (PTAL). For the relatively high‐frequency range relevant to speech perception in noise, we used PTA 1‐2‐4 kHz HL BC (PTAH). For the perception of ultrahigh sounds (e.g., music or nature), we investigated PTA 8‐10‐12.5 kHz SPL AC (PTAU). Missing audiometric data at the PTAs at baseline and after the first cisplatin cycle were imputed following the method described previously by Theunissen et al.,^26^ while patients with missing posttreatment audiometry were excluded.

### 2.3 Isolation of DNA and sequencing

DNA was isolated from FFPE tissue blocks at NKI. Next, the Princess Margaret Cancer Centre in Toronto performed sequencing of 31 SNPs by MassArray SNP genotype method (Sequenom) using Assay Design Suite software.

### 2.4 Gene selection

The genetic variants were selected based on published literature, had a minor allele frequency in individuals of European descent of at least 1%, and had the ability to incorporate the polymorphism or a surrogate in high (D' >0.95) linkage disequilibrium in the multiplex reaction. Included SNPs and associated references from literature review can be found in Table S1.

In our analysis, certain SNPs identified as significant in previous literature were not available in our genotyping panel; therefore, we selected proxy variants based on linkage disequilibrium data and prior association studies. Specifically, rs1051740 was used as a proxy for rs1142345, rs2273697 for rs1800462, and rs4646316 for rs11568591.

### 2.5 Statistical analysis

Descriptive statistics were used to summarize baseline patient characteristics and allele and genotype frequencies of the 31 SNPs of interest.

Multivariate imputation was performed using the panImpute function from the mitml package in R to account for missing data. Missing audiometry data at baseline (4 ears [3.5%] for PTAL and 4 ears [3.5%] for PTAH), and after the first cisplatin infusion (57 ears [49.6%] for PTAL, 57 ears [49.6%] for PTAH, and 31 ears [27.0%] for PTAU), were imputed. Imputations were functions of the outcome (i.e., posttreatment PTAs) as well as PTAs at baseline and first chemotherapy, chemotherapy dosage and cochlear radiation dose. PTAs at baseline, the first cisplatin infusion, and posttreatment were log‐transformed to ensure normally distributed residuals where necessary and back‐transformed for use in the main model of interest.

Quality control analysis was performed on the genetic data. This included the exclusion of SNPs without variation in allele frequency. The rs77382849 SNP (*EIF3A*) was excluded from the analysis, as all patients were observed to have the homozygous major genotype, rendering it uninformative for association analysis. Additionally, the Hardy–Weinberg equilibrium was assessed, and no SNPs violated the assumption (*p*‐value>0.05).

CIHL may differ in both ears, as the ear ipsilateral to the tumor may receive a higher dose of radiation compared with the contralateral ear. Thus, the outcome was expressed per ear on two occasions: left and right ears after the first cisplatin infusion and the left and right ears after the end of treatment. To determine if any of the candidate SNPs were independently associated with posttreatment hearing capability, linear mixed‐effects models on posttreatment PTA with patient‐specific intercepts were run for one SNP at a time.

We created two sets of models: ‘Unadjusted models’ evaluated the association of a SNP with posttreatment hearing capability adjusted for pretreatment high‐frequency PTA (PTAH) only. ‘Adjusted models’ evaluated the association of a SNP on the same posttreatment hearing capability adjusted for all pretreatment (low, high, and ultra‐high) PTAs (PTAL, PTAH, PTAU), cumulative chemotherapy and radiation dose to the cochlear. We adjusted for the same clinical factors as those used in the original model by Theunissen et al. To account for additive gene effects, the genotype with the major allele homozygous was coded as ‘0’, the heterozygous genotype as ‘1’, and the genotype with the minor allele homozygous as ‘2’. Pooling of results of 100 imputed datasets was performed using the summary function (testEstimates) from the mitml package. Unadjusted and adjusted *p*‐values were computed without correction for multiple testing; rather than dismissing associated SNPs to control family‐wise error rate, we included significant SNPs with a liberal *p*‐value threshold to preserve any potentially clinically important SNPs in the final model.

To ensure consistency in findings, a sensitivity analysis was performed using co‐dominant, adjusted linear mixed‐effects regression models. For this model, patients with two major alleles in a given gene were coded as zero, heterozygous patients were coded as 1, and patients with two minor alleles were coded as 2. We utilized the co‐dominant genetic model due to its flexibility and detailed categorization of genotypes.

We performed a likelihood ratio test (LRT) to determine the goodness of fit of the two models, with and without the SNP. Model comparisons were calculated and summarized over the 100 imputed datasets.

To evaluate and compare the performance of the new prediction model to predict observed PTAH of at least 35 dB de novo (the Dutch threshold for hearing aid qualification) to the original prediction model, we computed the 10‐fold cross‐validated sensitivity and specificity of both models at all possible thresholds of predicted HLs. Cross‐validation was performed at the patient level. Sensitivity and specificity for each cut‐off were imposed on the model predictions to classify patients as “hearing loss” (predicted hearing at/above cut‐off in either ear) versus “no hearing loss” (predicted hearing below cut‐off in both ears). Patients with missing data for any identified statistically significant SNP associated with the outcome, or those who had baseline PTAH ≥35 dB (therefore classified as “hearing loss” before treatment) were removed from cross‐validation. We then constructed the receiver operating characteristics (ROC) curve by plotting 10‐fold cross‐validated sensitivity versus 1‐specificity and calculated the area under the curve (AUC) with 95% confidence intervals. To assess the discriminatory performance of the models, we performed DeLong's test to compare the ROC curves of the new and the original prediction model to statistically evaluate and compare the predictive accuracy of the two models.

Data were analyzed using R software (v.2022.12.0). All tests were two‐sided and *α* = 0.05 was used to denote statistical significance.

## 3 RESULTS

### 3.1 Patient selection

A total of 115 HNSCC patients received high‐dose chemoradiation as a primary treatment and had genetic SNP and audiometry data available. If a patient had complete data for only one ear, that ear was retained for analysis. We excluded 35 ears with missing radiation dose to the cochlea (both ears from 17 patients, one ear from 1 patient), and 54 ears without posttreatment PTAH (both ears from 24 patients, one ear from 6 patients). In total, 74 patients (64.3%) and 141 ears were included (Figure S2). Due to the unavailability of tissue samples for some subjects from Theunissen et al.'s cohort, our current cohort differs slightly. Additionally, it includes 18 patients who underwent treatment with IA cisplatin.

### 3.2 Descriptive analysis

Table 1 summarizes the patient sample. The mean age of participants was 53.9 (SD = 8.5) years and 77% (*n* = 57) of participants were male. The cumulative cisplatin dose among patients ranged from 315 to 1200 (median = 579.5) mg. The radiation dose to the cochlea ranged from 1.1 to 70.5 Gy (median = 11.4 Gy) because the patients were, in general, treated with intensity‐modulated radiotherapy.

**TABLE 1:** Descriptive statistics of patient population (*n* = 74) demographics, chemotherapy, and radiation dose to the cochlea.

Variable	Value
No. of patients	74
Sex (%)	
Male	57 (77.0%)
Female	17 (23.0%)
Age, mean (SD), years	53.9 (8.5)
Tumor site (%)	
Oropharynx	45 (60.8%)
Hypopharynx	17 (23.0%)
Oral cavity	5 (6.8%)
Larynx	3 (4.1%)
Other head and neck sites	4 (5.5%)
Cumulative cisplatin dose, median (min, max), mg	579.5 (315.0, 1200.0)
Intra‐arterial cisplatin (%)	18 (24.3%)
Intravenous cisplatin (%)	56 (76.7%)
Cochlear radiation dose, median (min, max), Gy	11.4 (1.1, 70.5)

Abbreviations: Min, minimum; Max, maximum; SD, standard deviation.

Table S2 details allele and genotype frequency across the patient sample. Homozygous minor allele genotypes were not present for six SNPs (rs11085735 (*KEAP1*), rs12201199 (*TPMT*), rs316019 (*SLC22A2*), rs596881 (*SLC22A2/OCT2*), and rs2291767 and rs77124181 (*OTOS*)) and homozygous major allele genotype only was observed for one SNP (rs77382849 (*EIF3A*)), and therefore it was removed from analysis. Hence, 30 SNPs in total were included in the model building.

**FIGURE 1:**
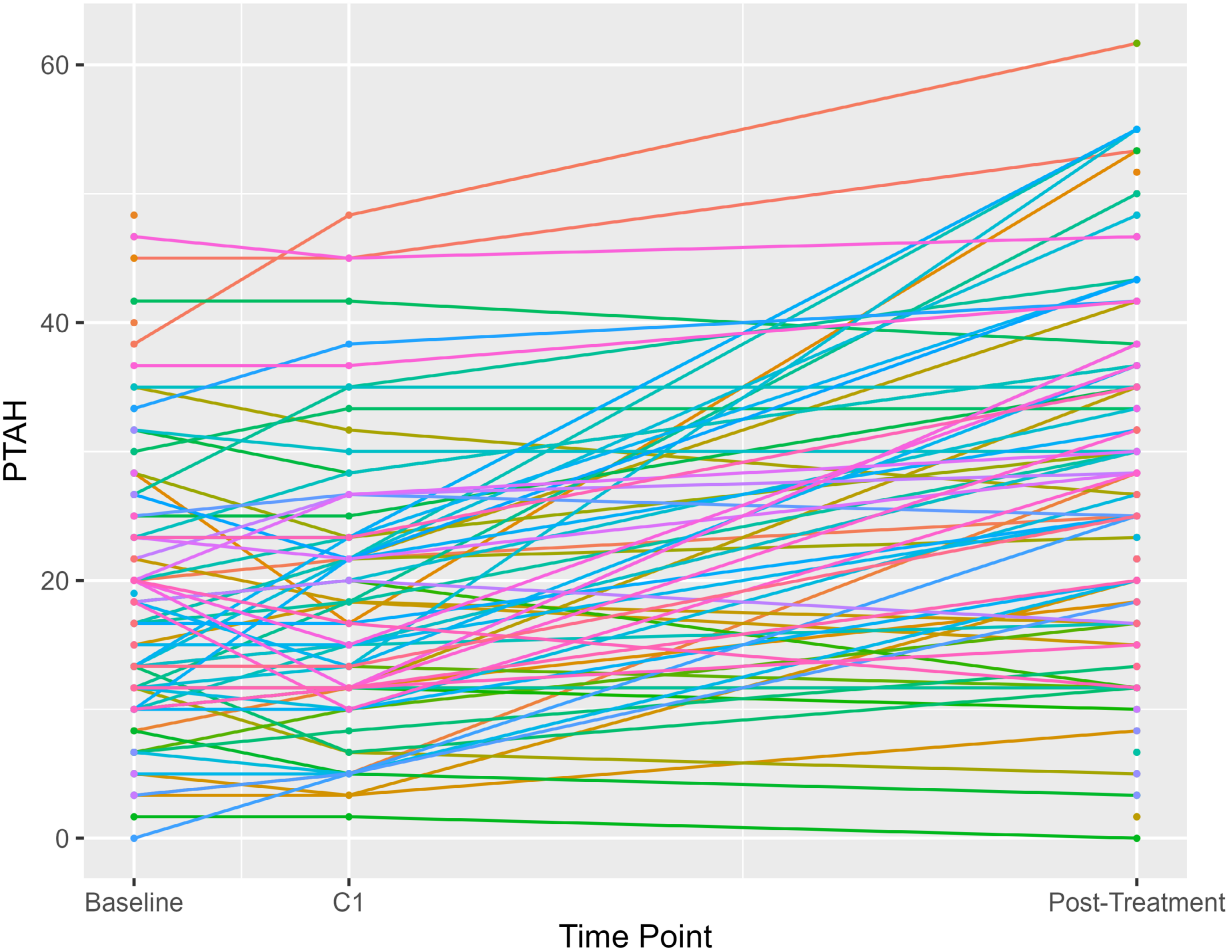
Spaghetti plot of pure tone audiometry at high frequencies (PTAH) in kHz, a measure of hearing loss, over time, from baseline to posttreatment. C1: first cisplatin dose. Time between baseline and C1 was 3 weeks, time between C1 and posttreatment was median 14 weeks.

For the majority of patients, as time progressed from the first cisplatin infusion to posttreatment, PTAH (kHz) increased over time (i.e., hearing loss occurred) (Figure 1).

### 3.3 Gene analysis

Of the 30 candidate SNPs, only one was a statistically significant predictor of posttreatment hearing capability. The rs2289669 SNP (within gene *SLC47A1/MATE1*) showed a 2.67 dB (95% CI 0.49–4.86, *p* = 0.017) greater hearing loss for each additional minor allele (Table 2).

**TABLE 2:** Unadjusted and adjusted additive, linear mixed‐effects regression model results for each of the 30 SNPs of interest.

SNP	Unadjusted model^b^			Adjusted model^c^		
	Coefficient^a^	95% CI	*p*‐value	Coefficient	95% CI	*p*‐value
rs1048290 *KEAP1*	−0.99	−3.17, 1.20	0.376	−0.6	−3.05, 1.84	0.629
rs1051640 *ABCC3*	−1.1	−3.71, 1.52	0.412	−1.27	−4.04, 1.5	0.37
rs1051740 *EPHX1*	−0.25	−2.86, 2.35	0.851	−0.01	−2.75, 2.73	0.995
rs10981694 *SLC31A1*	−1.98	−4.47, 0.51	0.118	−2.14	−4.77, 0.5	0.112
rs11085735 *KEAP1*	−1.35	−5.91, 3.21	0.561	−1.92	−6.81, 2.97	0.442
rs11615 *ERCC1*	0.14	−1.99, 2.26	0.9	0.16	−2.08, 2.4	0.888
rs12201199 *TPMT*	1.84	−2.51, 6.19	0.407	2.6	−2.05, 7.26	0.273
rs13181 *ERCC2*	−0.63	−2.83, 1.57	0.572	−0.31	−2.65, 2.02	0.791
rs1695 *GSTP1*	−1.31	−3.54, 0.91	0.247	−0.85	−3.22, 1.52	0.483
rs1801133 *MTHFR*	0.73	−1.42, 2.89	0.506	−0.37	−2.68, 1.94	0.752
rs1806649 *NFE2L2*	−0.87	−3.32, 1.57	0.483	−1.11	−3.68, 1.47	0.4
rs1872328 *ACYP2*	1.43	−2.49, 5.35	0.474	−0.16	−4.42, 4.1	0.942
rs2075252 *LRP2*	−0.71	−3.09, 1.67	0.559	−0.48	−3.05, 2.08	0.711
rs2228001 *XPC*	−0.3	−2.47, 1.87	0.787	0.2	−2.16, 2.55	0.87
rs2228171 *LRP2*	0.06	−2.13, 2.24	0.959	0.4	−1.92, 2.72	0.734
rs2273697 *ABCC2*	−1.84	−4.85, 1.16	0.229	−2.12	−5.31, 1.07	0.193
rs2289669* *SLC47A1*	2.96	0.92, 4.99	0.004	2.67	0.49, 4.86	0.017
rs2291767 *OTOS*	−3.99	−11.48, 3.51	0.297	−1.19	−9.32, 6.93	0.774
rs316019 *SLC22A2*	0.79	−3.02, 4.60	0.686	3.12	−0.92, 7.16	0.13
rs3212986 *ERCC1*	−0.01	−2.46, 2.44	0.993	0.28	−2.33, 2.88	0.835
rs3740066* *ABCC2*	2.14	−0.36, 4.63	0.093	2.4	−0.27, 5.06	0.078
rs4480 *SOD2*	1.28	−0.87, 3.44	0.244	0.93	−1.38, 3.24	0.431
rs4646316 *COMT*	−0.16	−2.82, 2.50	0.904	−1.38	−4.29, 1.52	0.351
rs4788863 *SLC16A5*	−1.05	−3.44, 1.34	0.388	−0.81	−3.37, 1.75	0.535
rs596881 *SLC22A2/OCT2*	1.31	−2.66, 5.28	0.519	3.27	−0.92, 7.46	0.126
rs62283056 *WFS1*	0.75	−1.99, 3.49	0.589	2.11	−0.79, 5.02	0.154
rs717620 *ABCC2*	0.29	−2.52, 3.09	0.842	0.43	−2.53, 3.38	0.777
rs77124181 *OTOS*	−2.54	−8.13, 3.05	0.373	−2.22	−8.12, 3.67	0.46
rs7851395 *SLC31A1*	0.36	−1.82, 2.54	0.748	0.72	−1.59, 3.03	0.541
rs9332377 *COMT*	0.62	−2.45, 3.69	0.692	0.07	−3.19, 3.32	0.968

* Significant or borderline significant SNPs.

^a^ Model coefficients are interpreted as the change in follow‐up PTAH for each additional minor allele. For example, each additional minor allele of the rs2289669 SNP was associated with a 2.67 dB increase in follow‐up PTAH Lower values of PTAH indicate better hearing capability.

^b^ Unadjusted models include adjustment for pretreatment PTAH only.

^c^ Adjusted models include adjustment for all pretreatment (low, high, and ultra‐high) PTAs (PTAL, PTAH, PTAU), chemotherapy dosage, and radiation dose to the cochlea.

### 3.4 Sensitivity analysis

In a sensitivity analysis, regression models were re‐analyzed using a co‐dominant model. The rs2289669 SNP (particularly the homozygous minor AA genotype) was again the only significant predictor of posttreatment hearing capability, consistent with our additive gene model findings (Table S3).

### 3.5 Model evaluation

The inclusion of the rs2289669 SNP to our preexisting clinical prediction model including baseline PTA, chemotherapy dose and radiation dose, significantly improved its goodness‐of‐fit (LRT *p* = 0.017). Sensitivity, specificity, positive predictive value (PPV) and negative predictive value (NPV) at different hearing cut‐offs for both models (“SNP model” with the SNP and “clinical‐only model” with clinical factors only) are displayed in Table 3. The addition of rs2289669 improved sensitivity and specificity at a threshold of 30 and 35 dB (sensitivity 0.44 vs. 0.38; specificity 0.96 vs. 0.94) but not at 25 and 40 dB.

**TABLE 3:** Sensitivity and specificity at different hearing cut‐offs (25–40 dB).

Clinical‐only model^a^					SNP model^b^			
Patients (*N* = 65), ears (*N* = 127)								
Cut‐off for determining predicted hearing loss (dB)	Sensitivity	Specificity	Positive predictive value (PPV)	Negative predictive value (NPV)	Sensitivity	Specificity	Positive predictive value (PPV)	Negative predictive value (NPV)
25	0.833	0.596	0.441	0.903	0.778	0.553	0.400	0.867
**30**	**0.611**	**0.766**	**0.500**	**0.837**	**0.722**	**0.787**	**0.565**	**0.881**
**35**	**0.389**	**0.936**	**0.700**	**0.800**	**0.444**	**0.957**	**0.800**	**0.818**
40	0.278	0.979	0.833	0.780	0.278	0.979	0.833	0.780

^a^ Clinical‐only model predicts hearing loss from baseline PTA, chemotherapy dose, and cochlear radiation dose.

^b^ SNP model predicts hearing loss from these factors plus rs2289669 genotype. Bolded rows are where the SNP model has improved performance over the clinical‐only model.

We performed a supplementary analysis to observe changes in predictive ability with the addition of the top 10 SNPs, chosen based on significance (i.e., *p*‐values). We found that adding 10 more SNPs to the model did not improve the predictive ability, and, due to substantial overfitting, resulted in a decrease in the AUC.

**FIGURE 2:**
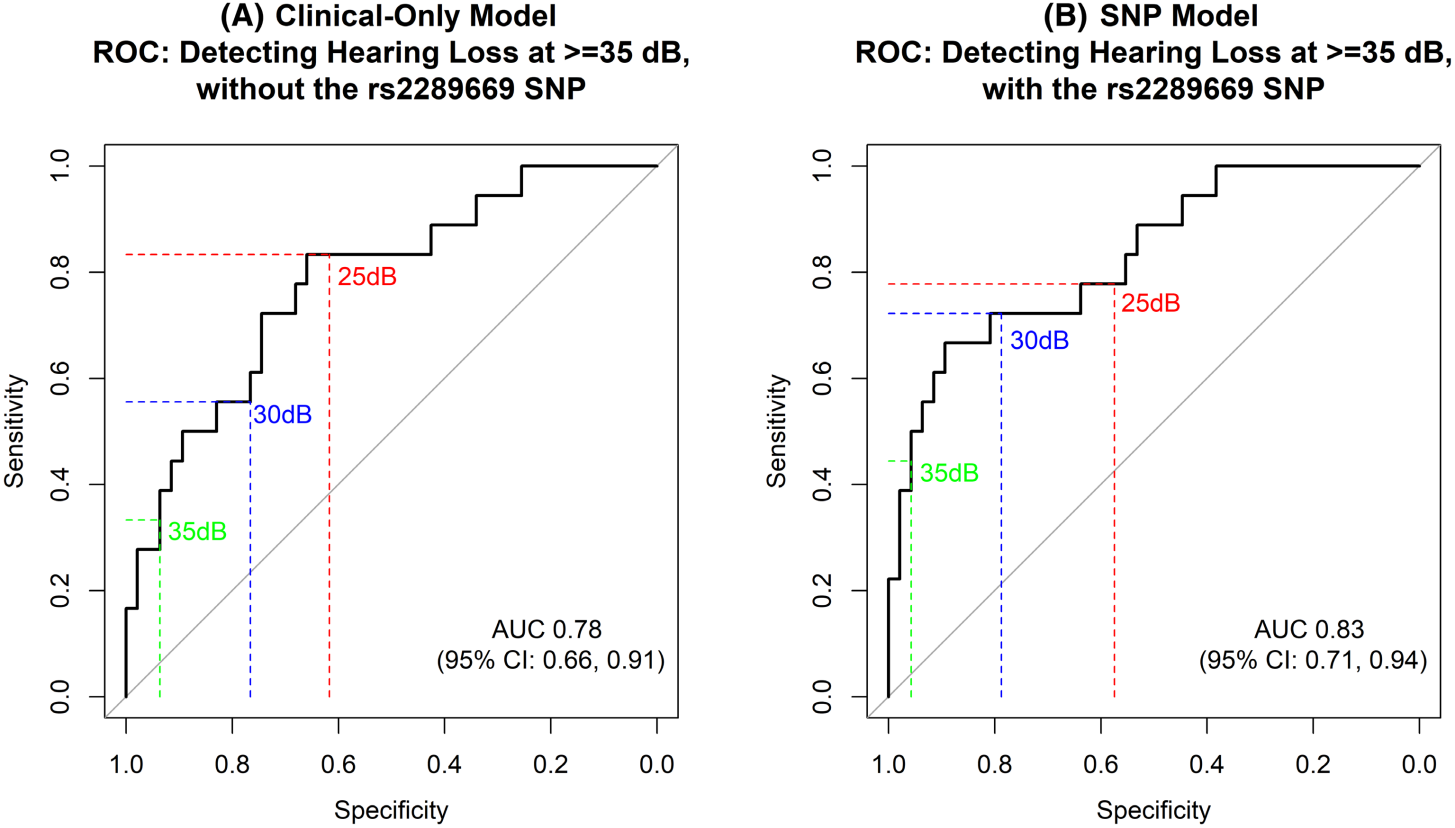
Receiver operating characteristics (ROC) curve for models with (SNP model) and without (clinical‐only model) incorporation of the rs2289669 SNP.

The SNP model had a moderately, borderline significant (*p* = 0.073), higher AUC (AUC = 0.83, 95% CI 0.71–0.94) compared to the clinical‐only model (AUC = 0.78, 95% CI 0.66–0.91). The observed predictive gains were not consistent across all hearing thresholds, with the SNP model demonstrating the greatest advantage at the 30 dB HL, as evident from the ROC curve plots (Figure 2).

## 4 DISCUSSION

This study aimed to enhance the predictive ability of the clinical prediction model for CIHL designed by Theunissen et al. by adding genetic information. We examined the association between 30 SNPs and posttreatment hearing capacity in HNSCC patients who received combined chemoradiation. Our gene analysis indicated that one SNP, rs2289669 within the *SLC47A1*/Multidrug and toxin extrusion 1 (*MATE1*) gene, significantly predicted posttreatment hearing capability. Our findings suggests that individuals with less frequent variants of this SNP (i.e., homozygous minor) are more likely to develop CIHL, compared to those with more common variants. For context, in our cohort, 17.8% of individuals carried the homozygous minor allele for rs2289669, highlighting the potential impact of these rarer variants. When the rs2289669 SNP was incorporated into the previously designed clinical prediction model, it showed enhanced predictive power. Although predictive gains were not consistent across all hearing thresholds, adding the SNP improved the clinical predictive model at key HLs and thresholds, namely 35 dB at PTA 1‐2‐4 kHz, relevant for the perception of speech and reimbursement of hearing aids in the Netherlands.

Genetic variations in cellular transporters may explain individual vulnerabilities to cisplatin‐related toxicity. Recently, the role of *MATE1* in the development of cisplatin toxicity was studied.^29^, ^30^, ^31^, ^32^ *MATE1* is an H+‐coupled organic cation bidirectional antiporter, expressed on the apical membrane of the tubular epithelium of the kidneys.^25^, ^32^ The human tissue distribution of *MATE1* is comparable to that in mice, where it has been shown to play an important role in the pharmacokinetics of several drugs.^25^ Nakamura et al. found that the plasma and renal levels of cisplatin were significantly higher in *MATE1* knock‐out mice than in wild‐type mice.^31^ *MATE1* likely interacts with the organic cation transporter 2 (*OCT2*) in the systemic distribution of cisplatin.^29^, ^32^ *OCT2* was already reported to be expressed in the cochlea and to be involved in the cochlear uptake of platinum. Interestingly, very recently, Waissbluth et al. showed that *MATE1* is expressed in the inner ear, and that cisplatin decreases both *OCT2* and *MATE1* expression in the cochlea.^30^

What is known about the *MATE1* rs2289669 variant from a study with patients using metformin—of which the uptake is also regulated by *MATE1*—is that it is likely associated with reduced transporter function.^33^ A mice study demonstrated that *MATE1* deficiency leads to increased cisplatin‐related nephrotoxicity and hematological toxicity (OR = 1.92, 95% CI 1.13 to 3.25, *p* = 0.016).^32^ Hence, if *MATE1* rs2289669 is indeed associated with reduced transporter function, this variant may also be associated with a higher risk of CIHL. This is supported by our results, as we found that the A‐allele is associated with more hearing loss. However, this contradicts Teft et al.'s findings that the rs2289669 *MATE1* A/A homozygous variant significantly reduces CIHL risk in 206 HNSCC patients (HR = 0.46, 95% CI 0.26–0.84).^25^ The exact role of *MATE1* in the cochlea needs to be further studied, although this may be challenging due to its bidirectional properties.^25^

Our analysis did not show significant associations between CIHL and several SNPs (*TPMT*,^23^ *ABCC3*,^23^, ^24^ *COMT*,^24^, ^25^, ^34^ *WFS1*,^18^, ^22^ *ACYP2*,^18^, ^19^, ^20^, ^21^, ^22^, ^34^, ^35^, ^36^ *ERCC2*,^34^ *XPC*,^34^ and *GSTP1* ^34^) as previously reported. The lack of consistency and reproducibility in study results, along with heterogeneity in study populations (in terms of ethnicity/ancestry, cisplatin treatment protocols, and definition of CIHL), contribute to the challenges in drawing definitive conclusions.^13^, ^20^ Additionally, many studies did not assess ultrahigh frequencies (8.0–20.0 kHz SPL), which generally are more sensitive frequencies for identifying CIHL, considering its initial manifestation at these frequencies.

This study benefits from a well‐defined cohort of patients who received combined chemoradiation as a primary treatment for HNSCC. The inclusion of SNPs and audiometry data allowed for a comprehensive analysis of the relationship between genetic variants and CIHL. Our study adopted a targeted approach by including 30 candidate SNPs in the model‐building process, strategically focusing on specific genetic markers of interest, based on published literature.

A limitation of the current study is that it relied on a retrospective analysis. Information on chemo‐ and radiotherapy dosage, audiometry, and other clinical factors was missing in a small number of patients. To address potential data gaps, we employed a mechanism of multiple imputation based on chained equations. Second, the selection of the 31 SNPs in this study was informed by a review of literature available up until 2017. Therefore, any genetic markers linked to ototoxicity discovered post 2017 were not included in our analysis. This could mean that potentially relevant SNPs identified after this cutoff were not included in the analysis. Finally, the predictive ability of the model, despite the inclusion of genetic variants, still demonstrated limitations in sensitivity and specificity. This suggests that factors beyond the current set of variables, such as environmental factors or additional genetic markers, may contribute to the variability in CIHL susceptibility.

CIHL is a common and debilitating side effect of cisplatin treatment, with important consequences on patients' quality of life.^8^ Understanding the genetic factors contributing to CIHL may enhance clinical prediction models, allowing clinicians to provide tailored pretreatment counseling, inform patients about the potential risk of CIHL, and enable the identification of individuals who may benefit from otoprotectants. Moreover, the identification of SNPs related to CIHL may facilitate the development of novel preventive strategies and interventions. Additional research is needed to assess the validity and generalizability of the prediction model in various genetic backgrounds and clinical settings in other HNSCC patient populations. Furthermore, the specific mechanisms through which these genetic variants contribute to the development of CIHL need to be elucidated.

## 5 CONCLUSION

Our findings demonstrate a significant association between the rs2289669 SNP within the *SLC47A1* (*MATE1*) gene and posttreatment hearing capability in patients undergoing combined chemoradiation. These results contribute to a deeper understanding of the genetic factors influencing hearing outcomes in this population and provide insights into potential strategies for personalized treatment approaches in the future.

## AUTHOR CONTRIBUTIONS

**Charlotte W. Duinkerken:** Conceptualization (lead); data curation (lead); investigation (equal); writing – original draft (supporting); writing – review and editing (lead). **Sabrina Chiodo:** Conceptualization (supporting); data curation (supporting); formal analysis (lead); investigation (equal); methodology (equal); software (lead); validation (lead); visualization (lead); writing – original draft (lead); writing – review and editing (lead). **Katrina Hueniken:** Conceptualization (equal); data curation (equal); formal analysis (equal); investigation (equal); methodology (equal); software (equal); supervision (equal); validation (equal); visualization (equal); writing – original draft (equal); writing – review and editing (equal). **Michael Hauptmann:** Conceptualization (equal); methodology (supporting); writing – review and editing (equal). **Katarzyna Jóźwiak:** Conceptualization (equal); methodology (equal); writing – review and editing (equal). **Dangxiao Cheng:** Conceptualization (equal); writing – review and editing (equal). **Andrew Hope:** Conceptualization (equal); writing – review and editing (equal). **Geoffrey Liu:** Conceptualization (equal); data curation (equal); funding acquisition (lead); investigation (equal); methodology (equal); project administration (lead); supervision (lead); writing – original draft (equal); writing – review and editing (equal). **Charlotte L. Zuur:** Conceptualization (equal); data curation (equal); investigation (equal); methodology (equal); supervision (lead); writing – original draft (equal); writing – review and editing (equal).

## FUNDING INFORMATION

This study was supported by the Princess Margaret Cancer Centre Head & Neck Translational Program, with philanthropic funds from the Wharton Family, Joe's Team, and Gordon Tozer.

## CONFLICT OF INTEREST STATEMENT

The authors declare no competing interests.

### ETHICS STATEMENT

The study was approved by the ethics committee of The Netherlands Cancer Institute, and written informed consent was obtained from all patients before treatment.

## Supporting information


Data S1.

## Data Availability

Research data are not shared due to privacy and ethical considerations. Consent for analysis was granted exclusively to our research team by the Netherlands Cancer Institute. Requests for further information should be directed to the Netherlands Cancer Institute, but please note that the data cannot be shared outside of the specific consent parameters provided by the participants.
