# Supplementary material for: The role of genetic variants in the prediction of hearing loss due to cisplatin chemoradiotherapy
Source: Cancer Med. 2024 Aug 19;13(16):e7465. doi: 10.1002/cam4.7465 (PMC11332395; doi:10.1002/cam4.7465)
Supplement: Supplementary file 1 — Data S1. [file CAM4-13-e7465-s001.docx]

**Online-Only Supplement**

Items included in the document:

**eTable 1. Included SNPs and associated references from literature review.**

**eTable 2. Included SNPs and associated references from literature review.**

**eTable 3. Included SNPs and associated references from literature review.**

**eFigure 1. The prediction formula by Theunissen et al.**

**eFigure 2. Included SNPs and associated references from literature review.**

**eTable 1. Included SNPs and associated references from literature review.**

| **Gene** | **SNP** | **Source** |  |
| --- | --- | --- | --- |
|  |  |  |  |
| *KEAP1* | rs1048290 | Zazuli et al. (2018) |  |
| *ABCC3* | rs1051640 | Pussegoda et al. (2013); Wheeler et al. (2017); Spracklen et al. (2017) |  |
| *EPHX1* | rs1051740  *(proxy for rs1142345)* | Teft et al. (2019) |  |
| *SLC31A1* | rs10981694 | Teft et al. (2019) |  |
| *KEAP1* | rs11085735 | Zazuli et al. (2018) |  |
| *ERCC1* | rs11615 | Caronia et al. (2009); Lopes-Aguiar et al. (2017) |  |
| *TPMT* | rs12201199 | Teft et al. (2019) |  |
| *ERCC2* | rs13181 | Caronia et al. (2009); Lopes-Aguiar et al. (2017); Lui et al. (2018) |  |
| *GSTP1* | rs1695 | Pussegoda et al. (2013); Ross et al. (2009); Talach et al. (2016) |  |
| *MTHFR* | rs1801133 | Zazuli et al. (2018) |  |
| *NFE2L2* | rs1806649 | Zazuli et al. (2018) |  |
| *ACYP2* | rs1872328 | Teft et al. (2019) |  |
| *LRP2* | rs2075252 | Ross et al. (2009); Choeyprasert et al. (2013); Riedemann et al. (2008) |  |
| *XPC* | rs2228001 | Caronia et al. (2009); Lopes-Aguiar et al. (2017) |  |
| *LRP2* | rs2228171 | Ross et al. (2009); Choeyprasert et al. (2013); Riedemann et al. (2008) |  |
| *ABCC2* | rs2273697  *(proxy for rs1800462)* | Teft et al. (2019) |  |
| *SLC47A1/MATE1* | rs2289669 | Teft et al. (2019) |  |
| *OTOS* | rs2291767 | Spracklen et al. (2017) |  |
| *SLC22A2* | rs316019 | Teft et al. (2019) |  |
| *ERCC1* | rs3212986 | Caronia et al. (2009); Lopes-Aguiar et al. (2017) |  |
| *ABCC2* | rs3740066 | Teft et al. (2019) |  |
| *SOD2* | rs4480 | Brown et al. (2015) |  |
| *COMT* | rs4646316  *(proxy for rs11568591)* | Teft et al. (2019) |  |
| *SLC16A5* | rs4788863 | Drögemöller et al. (2018) |  |
| *SLC22A2* | rs596881 | Lanvers-Kaminsky et al. (2017), Spracklen et al. (2017); Wheeler et al. (2017) |  |
| *WFS1* | rs62283056 | Wheeler et al. (2017); Drögemöller et al. (2018) |  |
| *ABCC2* | rs717620 | Teft et al. (2019) |  |
| *EIF3A* | rs77382849 | Xu et al. (2012) |  |
| *OTOS* | rs77124181 | Spracklen et al. (2017) |  |
| *SLC31A1* | rs7851395 | Lanvers-Kaminsky et al. (2017); Xu et al. (2012) |  |
| *COMT* | rs9332377 | Teft et al. (2019) |  |

**Post-treatment hearing capability at a PTA of 1-2-4 kHz =**

**−5.56 + (0.02 × C) + (0.21 × RT) + (0.05 × PTAL) + (0.68 × PTAH) + (0.10 × PTAU)**

C: cisplatin dose in milligrams;

PTAH: high pretreatment PTA of 1-2-4 kHz BC in decibels;

PTAL: low pretreatment PTA of 0.5-1-2 kHz BC in decibels;

PTAU: ultrahigh pretreatment PTA of 8-10-12.5 kHz AC in decibels;

RT: the radiation dose in Gray.

**eFigure 1. The prediction formula by Theunissen et al.**

The formula by Theunissen et al. ^27^ for predicting post-treatment hearing capability at a PTA of 1-2-4 kHz.


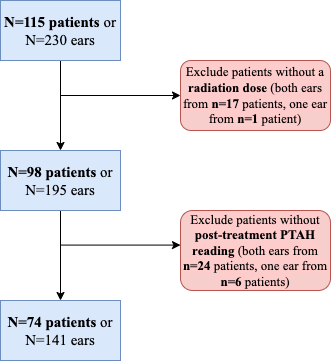


**eFigure 2. Exclusion flowchart detailing the number of patients and ears at each step of exclusion.**

**eTable 2. Minor allele and genotype frequency across patient population (n=74) for 31 SNPs of interest.**

| **Gene** | **SNP** | **Minor Allele** | | |
| --- | --- | --- | --- | --- |
|  |  | **Allele** | **Frequency** | **Percent (%)** |
| *KEAP1* | rs1048290 | C | 65/73 | 44.5 |
| *ABCC3* | rs1051640 | G | 29/73 | 19.9 |
| *EPHX1* | rs1051740 | C | 47/73 | 32.2 |
| *SLC31A1* | rs10981694 | G | 26/72 | 17.8 |
| *KEAP1* | rs11085735 | A | 9/73 | 6.2 |
| *ERCC1* | rs11615 | G | 59/73 | 40.4 |
| *TPMT* | rs12201199 | A | 11/73 | 7.5 |
| *ERCC2* | rs13181 | G | 50/72 | 34.2 |
| *GSTP1* | rs1695 | G | 51/73 | 34.9 |
| *MTHFR* | rs1801133 | A | 48/73 | 32.9 |
| *NFE2L2* | rs1806649 | T | 41/73 | 28.1 |
| *ACYP2* | rs1872328 | A | 10/73 | 6.8 |
| *LRP2* | rs2075252 | T | 41/69 | 28.1 |
| *XPC* | rs2228001 | G | 66/73 | 45.2 |
| *LRP2* | rs2228171 | T | 45/73 | 30.8 |
| *ABCC2* | rs2273697 | A | 31/68 | 21.2 |
| *SLC47A1/MATE1* | rs2289669 | A | 63/73 | 43.2 |
| *OTOS* | rs2291767 | C | 3/73 | 2.1 |
| *SLC22A2* | rs316019 | A | 15/73 | 10.3 |
| *ERCC1* | rs3212986 | A | 38/73 | 26.0 |
| *ABCC2* | rs3740066 | T | 52/72 | 35.6 |
| *SOD2* | rs4480 | T | 51/73 | 34.9 |
| *COMT* | rs4646316 | T | 32/73 | 21.9 |
| *SLC16A5* | rs4788863 | T | 39/73 | 26.7 |
| *SLC22A2* | rs596881 | T | 13/73 | 8.9 |
| *WFS1* | rs62283056 | C | 32/73 | 21.9 |
| *ABCC2* | rs717620 | T | 29/73 | 19.9 |
| *EIF3A** | rs77382849* | T | 0/73 | 0.0 |
| *OTOS* | rs77124181 | C | 6/73 | 4.1 |
| *SLC31A1* | rs7851395 | G | 60/72 | 41.1 |
| *COMT* | rs9332377 | T | 26/73 | 17.8 |

*Removed from further analysis as there were no minor alleles identified.

**eTable 3.** Unadjusted and adjusted co-dominant, linear mixed-effects regression model outputs for each of the 30 SNPs of interest.

| **SNP** | | **Unadjusted Models^a^** | | | **Adjusted Models^b^** | | |
| --- | --- | --- | --- | --- | --- | --- | --- |
|  |  | **PTAH Estimate** | **95% CI** | **p-value** | **PTAH Estimate** | **95% CI** | **p-value** |
| rs1048290 (*KEAP1*)  Ref: GG | GC | -0.23 | -3.69,3.24 | 0.90 | -0.56 | -4.4,3.27 | 0.77 |
|  | CC | -2.17 | -6.63,2.29 | 0.34 | -1.20 | -6.17,3.77 | 0.64 |
| rs1051640  (*ABCC3*)  Ref: AA | GA | -0.42 | -3.85,3.01 | 0.81 | -0.65 | -4.38,3.08 | 0.73 |
|  | GG | -3.63 | -10.62,3.36 | 0.31 | -3.76 | -11.12,3.59 | 0.32 |
| rs1051740  (*EPHX1*)  Ref: TT | TC | -0.10 | -3.29,3.08 | 0.95 | -0.23 | -3.61,3.15 | 0.89 |
|  | CC | -0.82 | -7.54,5.9 | 0.81 | 0.44 | -6.57,7.46 | 0.90 |
| rs10981694  (*SLC31A1*)  Ref: TT | GT | -1.20 | -4.81,2.4 | 0.51 | -1.36 | -5.16,2.45 | 0.48 |
|  | GG | -5.00 | -11.1,1.1 | 0.11 | -5.33 | -11.82,1.17 | 0.11 |
| rs11085735  (*KEAP1*)  Ref: CC | CA | -1.35 | -5.91,3.21 | 0.56 | -1.92 | -6.81,2.97 | 0.44 |
|  | AG | 3.37 | 0.06,6.68 | 0.05 | 3.03 | -0.48,6.55 | 0.09 |
| rs11615  (*ERCC1*)  Ref: AA | GG | -0.78 | -4.97,3.41 | 0.72 | -0.64 | -5.1,3.82 | 0.78 |
| rs12201199  (*TPMT*)  Ref: AA | TA | 1.84 | -2.51,6.19 | 0.41 | 2.60 | -2.05,7.26 | 0.27 |
| rs13181  (*ERCC2*)  Ref: TT | GT | -1.03 | -4.33,2.28 | 0.54 | -1.66 | -5.15,1.83 | 0.35 |
|  | GG | -0.99 | -5.75,3.76 | 0.68 | 0.41 | -4.65,5.47 | 0.87 |
| rs1695  (*GSTP1*)  Ref: AA | AG | -1.40 | -4.65,1.85 | 0.40 | -0.73 | -4.22,2.77 | 0.68 |
|  | GGA | -2.56 | -7.45,2.32 | 0.30 | -1.79 | -6.96,3.38 | 0.50 |
| rs1801133  (*MTHFR*)  Ref: GG | AG | 1.71 | -1.58,4.99 | 0.31 | 1.49 | -2.07,5.05 | 0.41 |
|  | A | 0.70 | -4.01,5.41 | 0.77 | -2.18 | -7.2,2.84 | 0.39 |
| rs1806649  (*NFE2L2*)  Ref: CC | CT | -0.79 | -4.01,2.43 | 0.63 | -1.27 | -4.69,2.14 | 0.46 |
|  | TT | -1.90 | -7.99,4.19 | 0.54 | -1.89 | -8.42,4.63 | 0.57 |
| rs1872328  (*ACYP2*)  Ref: GG | GA | 0.94 | -3.96,5.84 | 0.71 | -0.14 | -5.38,5.09 | 0.96 |
|  | AA | 4.63 | -8.03,17.29 | 0.47 | -0.37 | -14.33,13.58 | 0.96 |
| rs2075252  (*LRP2*)  Ref: CC | CT | 0.47 | -2.88,3.82 | 0.78 | -0.21 | -3.81,3.39 | 0.91 |
|  | TT | -2.73 | -8.18,2.71 | 0.33 | -1.30 | -7.27,4.67 | 0.67 |
| rs2228001  (*XPC*)  Ref: TT | GT | -0.79 | -4.41,2.84 | 0.67 | -0.49 | -4.32,3.33 | 0.80 |
|  | GG | -0.50 | -4.89,3.89 | 0.82 | 0.55 | -4.22,5.32 | 0.82 |
| rs2228171  (*LRP2*)  Ref: CC | CT | 0.27 | -3.08,3.62 | 0.87 | 0.29 | -3.24,3.82 | 0.87 |
|  | TT | -0.06 | -4.87,4.75 | 0.98 | 0.90 | -4.29,6.08 | 0.73 |
| rs2273697  (*ABCC2*)  Ref: GG | GA | -1.50 | -4.76,1.76 | 0.37 | -1.55 | -5,1.91 | 0.38 |
|  | AA | -7.21 | -19.83,5.41 | 0.26 | -10.01 | -23.65,3.63 | 0.15 |
| rs2289669*  (*SLC47A1/MATE1*)  Ref: GG | GA | 2.12 | -1.13,5.36 | 0.20 | 1.35 | -2.11,4.81 | 0.44 |
|  | AA | 6.26 | 2.07,10.44 | 0.005 | 5.88 | 1.39,10.36 | 0.01 |
| rs2291767  (*OTOS*)  Ref: TT | TC | -3.99 | -11.48,3.51 | 0.30 | -1.19 | -9.32,6.93 | 0.77 |
| rs316019  (*SLC22A2*)  Ref: CC | CA | 0.79 | -3.02,4.6 | 0.69 | 3.12 | -0.92,7.16 | 0.13 |
| rs3212986  (*ERCC1*)  Ref: CC | CA | 0.34 | -2.86,3.54 | 0.84 | 0.58 | -2.8,3.96 | 0.74 |
|  | AA | -0.67 | -6.88,5.54 | 0.83 | -0.01 | -6.7,6.67 | 1.00 |
| rs3740066  (*ABCC2*)  Ref: CC | TC | 2.51 | -0.77,5.79 | 0.13 | 2.34 | -1.16,5.84 | 0.19 |
|  | TT | 3.82 | -1.88,9.51 | 0.19 | 4.87 | -1.19,10.92 | 0.12 |
| rs4480  (*SOD2*)  Ref: CC | TC | 2.53 | -0.69,5.74 | 0.12 | 2.15 | -1.32,5.63 | 0.22 |
|  | TT | 1.63 | -3.05,6.31 | 0.50 | 0.99 | -4.01,5.99 | 0.70 |
| rs4646316  (*COMT*)  Ref: CC | CT | -2.00 | -5.16,1.15 | 0.21 | -2.47 | -5.88,0.95 | 0.16 |
|  | TT | 5.41 | -2.13,12.96 | 0.16 | 1.11 | -7.35,9.58 | 0.80 |
| rs4788863  (*SLC16A5*)  Ref: CC | CT | -1.44 | -4.77,1.9 | 0.40 | -1.06 | -4.62,2.49 | 0.56 |
|  | TT | -1.58 | -7.26,4.11 | 0.59 | -1.25 | -7.51,5.02 | 0.70 |
| rs596881  (*SLC22A2*)  Ref: CC | TC | 1.31 | -2.66,5.28 | 0.52 | 3.27 | -0.92,7.46 | 0.13 |
| rs62283056  *WFS1*)  Ref: G | GC | 1.16 | -1.96,4.28 | 0.46 | 2.52 | -0.77,5.81 | 0.13 |
|  | CC | -0.62 | -9.96,8.71 | 0.90 | 2.07 | -7.74,11.88 | 0.68 |
| rs717620  (*ABCC2*)  Ref: CC | TC | 1.11 | -2.15,4.36 | 0.50 | 0.58 | -2.87,4.03 | 0.74 |
|  | TT | -3.24 | -12.73,6.24 | 0.50 | 0.15 | -9.99,10.29 | 0.98 |
| rs77124181  (*OTOS*)  Ref: GG | CG | -2.54 | -8.13,3.05 | 0.37 | -2.22 | -8.12,3.67 | 0.46 |
| rs7851395  (*SLC31A1*)  Ref: AA | AG | 1.35 | -2.11,4.81 | 0.44 | 1.25 | -2.45,4.96 | 0.51 |
|  | GG | 0.34 | -4.12,4.8 | 0.88 | 1.24 | -3.51,5.99 | 0.61 |
| rs9332377  (*COMT*)  Ref: CC | TC | 1.03 | -2.33,4.38 | 0.55 | 0.63 | -2.91,4.18 | 0.73 |
|  | TT | -2.51 | -15.25,10.23 | 0.70 | -5.17 | -18.77,8.43 | 0.46 |

*Significant SNP

**^a^ Unadjusted models** include adjustment for pre-treatment PTAH only.

**^b^Adjusted models** include adjustment for all pre-treatment (low, high, and ultra-high) PTAs (PTAL, PTAH, PTAU), chemotherapy dosage, and radiation dose to the cochlea.
